# Gut microbiome disparities reflect type 2 diabetes progression and medication status

**DOI:** 10.1016/j.isci.2026.115156

**Published:** 2026-02-26

**Authors:** Manoj Kumar, Samradhi Singh, Raj Ojha, Mona Kriti, Gwoncheol Park, Vinod Verma, Namrata Pal, Poonam Sharma, Swasti Shubham, Megha K. Pandey, Devraj J. Parasannanavar, Devojit K. Sarma, Rajnarayan R. Tiwari, Ravinder Nagpal

**Affiliations:** 1 National Institute for Research in Environmental Health, Indian Council for Medical Research, Bhopal 462030, Madhya Pradesh, India; 2 The Gut Biome Lab, Florida State University, Tallahassee, FL 32306, USA; 3 Department of Health, Nutrition, and Food Sciences, College of Education, Health, and Human Sciences, Florida State University, Tallahassee, FL 32306, USA; 4 Translational Medicine Center, All India Institute of Medical Sciences, Bhopal, Madhya Pradesh 462020, India; 5 Division of Clinical Epidemiology, Indian Council for Medical Research-National Institute of Nutrition, Hyderabad 500007, India; 6 Stem Cell Research Centre, Department of Hematology, Sanjay Gandhi Post-Graduate Institute of Medical Sciences, Lucknow, Uttar Pradesh 226014, India

**Keywords:** endocrinology, microbiome, public health

## Abstract

Type 2 diabetes mellitus (T2D) prevalence is rapidly increasing in India, yet microbiome signatures linked to disease progression and oral antidiabetic therapy remain underexplored. We performed full-length 16S rRNA sequencing of fecal samples from prediabetics (PDs), untreated newly diagnosed T2D (UKT2D), and clinically diagnosed T2D patients (KT2D), alongside biochemical, anthropometric, and medication data. Despite comparable glycemic control and insulin resistance between UKT2D and KT2D groups, gut microbial diversity was significantly reduced in KT2D, coinciding with antidiabetic drug use, primarily metformin. *Lactobacillus* abundance increased with disease progression, while Clostridium_sensu_stricto_1 was associated with glucose homeostasis and insulin sensitivity. β diversity differed only between controls and PD, with no other pairwise differences. Collectively, these results indicate that T2D progression and oral antidiabetic medications remodel the gut microbiome in this south Asian cohort and highlight the need to reassess antidiabetic treatment efficacy using larger longitudinal studies.

## Highlights

• Gut microbiome signatures of T2D progression and treatment remain underexplored in India
• Full-length 16S rRNA sequencing profiled gut microbiomes across glycemic stages toward T2D
• Gut microbiome composition differs across disease stages and medication use
• Key glycemic markers remain comparable between untreated and treated T2D patients

## Graphical abstract

**Figure:**
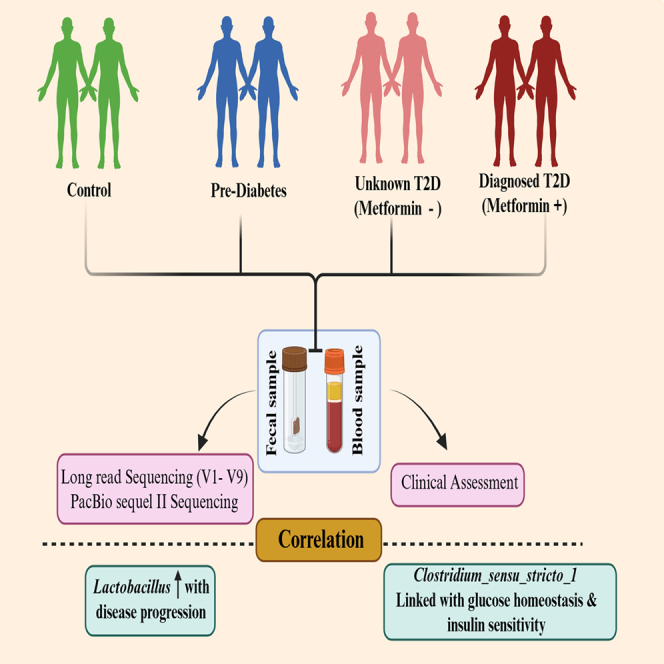

## Introduction

Type 2 diabetes mellitus (T2D) is a multifaceted metabolic disorder characterized by disruptions in glucose homeostasis and lipid metabolism. Its impact reverberates globally, compromising the quality of life and contributing to premature mortality, making it a paramount global concern, with increasing prevalence worldwide from a current global load of ∼537 million to an estimate of ∼783 million by 2045. In India, T2D projections forecast a rise from ∼74 million existing cases to ∼125 million in 2045.^1^

Emerging evidence demonstrates the link between the gut microbiome in T2D pathophysiology, revealing associations with specific microbial taxa. Commonly reported findings indicate that genera such as *Bifidobacterium*, *Bacteroides*, *Faecalibacterium*, *Akkermansia*, and *Roseburia* share a negative association with disease severity, whereas *Ruminococcus*, *Fusobacterium*, and *Blautia* are positively correlated. Interestingly, the genus *Lactobacillus* shows the most variable results across studies.^2^^,^^3^ However, given the high variability of gut microbiome in different populations, ethnicities, and races, limited data exist pertaining to the relationship of gut microbiome with T2D while also taking into account the effect of anti-diabetic drug use in the south-Asian population, specifically for India. Furthermore, broad metrics of microbial communities, such as various diversity indices and the ratio of predominant gut microbial phyla i.e., bacteroidetes/firmicutes ratio, once proposed as a marker of metabolic disease, including T2D, is increasingly being disregarded due to inconsistent findings.^3^^,^^4^

Anti-diabetic drugs are known to modulate the gut microbiota composition and function, though the effects observed often differ between animal models and human studies. Conversely, baseline microbiota can influence the pharmacokinetics and pharmacodynamics of these drugs through various mechanisms.^5^^,^^6^ Among anti-diabetic medications, metformin, a widely prescribed biguanide, has been shown to promote the growth of specific bacterial genera such as *Akkermansia muciniphila*, *Escherichia*, and various *Bifidobacterium* species^7^^,^^8^^,^^9^^,^^10^^,^^11^^,^^12^^,^^13^^,^^14^ while reducing the abundance of other, reportedly *Intestinibacter*, *Romboutsia*, and *Peptostreptococcaceae*.^8^^,^^12^^,^^14^

To better comprehend these complex interactions amid anti-diabetic drugs, T2D status, and the gut microbiome, we herein longitudinally profiled the same in known T2D (KT2D), unknown T2D (UKT2D), pre-diabetics (PD), and Ctrl milieus. We further distinguished the clinical parameters between the defined categories while assessing the effectiveness of anti-diabetic drugs, and explored the shifts in the gut microbiome composition with disease progression with regards to the use of oral anti-diabetic medication. Understanding these relationships is clinically important and can lead to novel and efficient avenues for targeted and effective treatments for T2D. Furthermore, timely identification and monitoring of distinct microbial signatures linked to various stages of glucose dysregulation could help tailor personalized treatment strategies. In these milieus, the findings from this study also inform the development of novel therapies that modulate gut bacteria, ultimately improving metabolic health. This comprehensive approach is crucial for addressing the rising diabetes epidemic in India as well as in other demographics experiencing such prevalence.

## Results

### Distinct hallmarks of clinical parameters in subjects with different T2DM disease status

**Figure 1:**
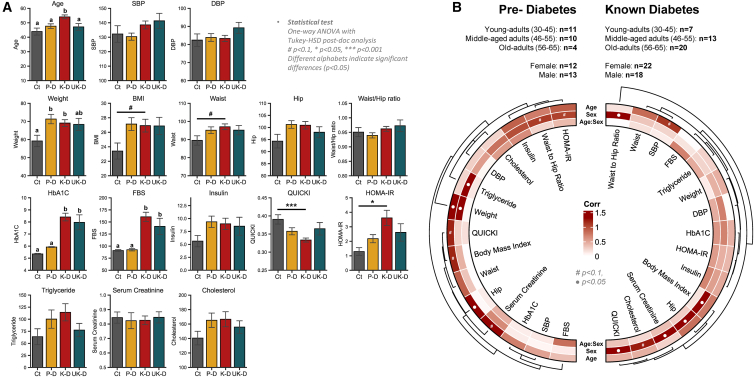
Distinct hallmarks of clinical parameters across cohorts of subjects (A) Bar plots representing clinical parameters across groups. Data are presented as mean ± SD. Statistical differences were determined using one-way ANOVA followed by Tukey’s HSD post hoc test. Different letters indicate statistical differences (*p* < 0.05). #*p* < 0.1; ∗*p* < 0.05; ∗∗∗*p* < 0.001. Ct, control; P-D, prediabetes; K-D, known-diabetes; Uk-D, unknown-diabetes. (B) Two-way ANOVA test examining the influence of age and sex on clinical parameters for pre-diabetes (left) and known-diabetes (right) patients. *p* values are –log transformed and used for visualization. The dendrogram is generated using hierarchical clustering results with the average linkage method, based on Bray-Curtis dissimilarity. “●” sign indicates *p* < 0.05.

To evaluate any significant changes in clinical parameters with disease progression, one-way ANOVA was performed followed by Tukey’s HSD test. Among the recorded clinical parameters such as BMI, cholesterol, systolic and diastolic blood pressure (SBP/DBP), FBS, HOMA-B, HOMA-IR, HbA1c, height, hip circumference, insulin, QUICKI, serum creatinine, triglycerides, waist circumference, WHtR, WHR, and weight, several parameters showed significant differences (Figure 1A). Specifically, age was significantly higher in individuals from the KT2D group, while weight was significantly lower in the Ctrl. HbA1c and FBS levels were significantly different in KT2D/UKT2D compared to PD/Ctrl at a 95% confidence interval (CI). QUICKI and HOMA-IR both exhibited strong differences between the KT2D and Ctrl groups. BMI and waist circumference differences were significant at a 90% CI between the KT2D and Ctrl groups. These findings suggest that the KT2D and UKT2D groups exhibit similar trends in these parameters implying whether diagnosed or undiagnosed may involve specific metabolic shifts not typically present in healthy individuals, and these shifts are more evident in KT2D individuals. Conversely, the PD group demonstrates similar trends in anthropometric measurements as KT2D and UKT2D, but its diabetes markers were more similar to those of the Ctrl group. Conducting a two-way ANOVA using age and sex as factors revealed significant differences in clinical parameters for each variable (Figure 1B). In the KT2D group, SBP changed with age at a 90% CI, while all other variables were non-significant. In contrast, sex as a factor showed significant changes at a 95% CI for WHR, BMI, hip circumference, serum creatinine, cholesterol, and QUICKI. In the PD group, age differentiated weight and hip circumference at a 95% CI, and QUICKI, BMI, and serum creatinine at a 90% CI. Meanwhile, sex affected triglycerides (TGs) and weight differentially at a 95% CI, and HOMA-IR and WHR at a 90% CI. However, there were no significant interactive effects between age and sex observed in either the PD or KT2D groups. In summary, this analysis highlights distinct metabolic and clinical parameter differences among healthy controls and individuals with KT2D, UKT2D, or PD. Age and sex independently affect these parameters, underscoring the importance of considering these factors in the clinical management of T2D and related conditions.

### Gut microbiome disparities in subjects with different stage of T2D progression

**Figure 2:**
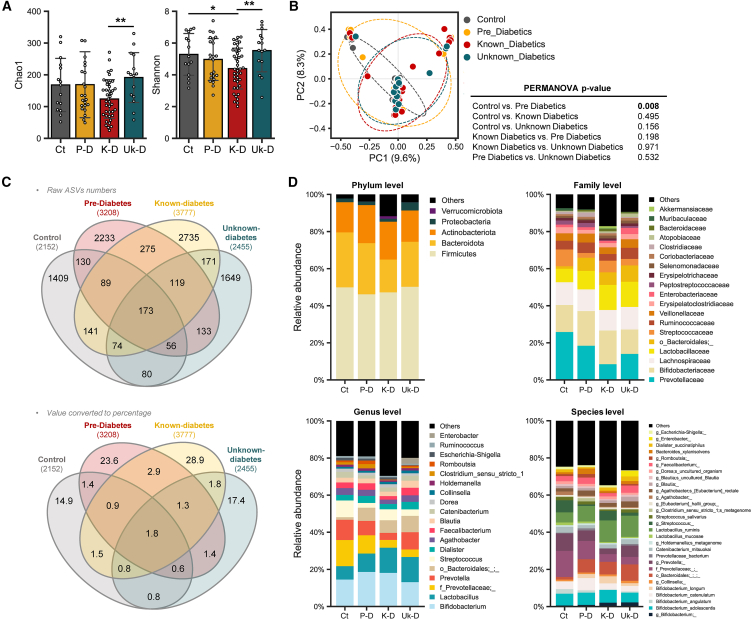
Gut microbiome disparities in subjects with different stage of T2D progression (A) Alpha-diversity is determined using the observed ASVs and Shannon indices. Significance between groups is calculated using the non-parametric Kruskal-Wallis test. (B) PCoA analysis based on Bray-Curtis dissimilarity calculated on ASVs is used to represent the beta-diversity of each group. Significance is calculated using PERMANOVA with 999 random permutations. (C) Venn diagrams showing the number (upper) or proportion (lower) of unique and shared ASVs among groups (ASV-based overlap is shown to provide a high-resolution overview of microbial community structure; biological interpretation and downstream analyses are based on taxonomic assignments). (D) Mean relative abundance of bacterial taxa across groups at specified taxonomic ranks. This panel summarizes group-level compositional trends.

Significant differences in microbial diversity among T2D groups were discerned through alpha-diversity indices, specifically Shannon and observed amplicon sequence variants (ASVs) (Figure 2A). The KT2D group exhibited a marked reduction in alpha-diversity across both indices compared to the other groups, especially UKT2D, indicating a distinctively different number and proportion of microbes. Regarding beta-diversity assessed by Bray-Curtis dissimilarity matrix, only the PD group showed a significant difference compared to the Ctrl group (PERMANOVA, *p* = 0.008) (Figure 2B). Overall, most samples clustered together regardless of group, forming the largest cluster, though some samples from KT2D and UKT2D groups clustered together, and some from the Ctrl group clustered distinctly, resulting in slight differences in overall sample distribution between Ctrl and KT2D/UKT2D. In contrast, samples from the PD group exhibited the most dispersed distribution among all groups, spanning all three clusters. These results indicate that some subjects from the KT2D and UKT2D groups shared microbial communities distinct from those of the Ctrl group, whereas subjects from the PD group shared microbial communities with all three groups.

To examine the microbial compositional characteristics and distinctions between groups, the number of unique and shared ASVs was assessed using Venn diagrams (Figure 2C). Contrasting with the microbial diversity, the KT2D group had the highest number of unique ASVs (n2735), which accounts for 28.9% of the total identified ASVs and 72.4% of the ASVs identified in the group. In contrast, the Ctrl and UKT2D groups showed relatively fewer unique ASVs: n1409 (14.9%, 65.5%) and n1649 (17.4%, 67.2%), respectively. This suggests that microbial composition varies among subjects in the KT2D group compared to other groups. Interestingly, the PD group exclusively shared the most ASVs with KT2D (n275 ASVs, 2.9% of the total identified ASVs), resulting in these two groups having the highest number of shared ASVs (6.9% of the total identified ASVs) compared to other group comparisons (ranging between 4.0% and 5.7%).

Subsequently, the relative microbial composition from phylum to species levels was calculated (Figure 2D). At the phylum level, *Firmicutes* emerged as the most dominant across all groups. The second most abundant phylum varied: *Actinobacteriota*, typically third in abundance, was second in the KT2D group, surpassing *Bacteroidota*. *Verrucomicrobiota*, which contains the *Akkermansia* genus (detection frequency [df], Obs. = 12%, KT2D = 18%, UKT2D = 13%, PD = 8%, Ctrl = 0.0%), exhibited a relative increase in abundance in the KT2D group compared to other groups. In the UKT2D group, *Proteobacteria* was more abundant compared to other groups. At the family level, *Prevotellaceae* was least abundant in KT2D relative to others, while an unknown family in the *Bacterioidales* order was least abundant in the Ctrl group. At the genus level, *Lactobacillus* (df, Obs. = 96%, KT2D = 100%, UKT2D = 100%, PD = 84%, Ctrl = 100%) abundance tended to increase with disease progression, while an unknown genus in *Prevotellaceae* family showed a reverse trend. *Streptococcus* (df, Obs. = 81%, KT2D = 73%, UKT2D = 87%, PD = 92%, Ctrl = 80%) was relatively less abundant in PD and UKT2D, increasing in KT2D and reaching its highest levels in the Ctrl group. *Clostridium_sensu_stricto_1* (df, Obs. = 56%, KT2D = 28%, UKT2D = 73%, PD = 76%, Ctrl = 80%) and *Romboutsia* (df, Obs. = 48%, KT2D = 18%, UKT2D = 67%, PD = 76%, Ctrl = 67%) were most abundant in the PD group, followed by the Ctrl group, and were relatively less in the KT2D and UKT2D groups. In addition, *Enterobacter* (df, Obs. = 6%, KT2D = 3%, UKT2D = 7%, PD = 12%, Ctrl = 7%) was highest in UKT2D. More specifically, at the species level, an unknown species in the *Bifidobacterium* genus (df, Obs. = 100%, KT2D = 100%, UKT2D = 100%, PD = 100%, Ctrl = 100%) and *B*. *longum* was relatively more abundant in KT2D and UKT2D, whereas *B*. *angulatum* was nearly depleted in these groups compared to Ctrl group. *Lactobacillus mucosae* was more abundant in the Ctrl group compared to other groups.

To further investigate the changes in microbiome composition between experimental groups and identify potential biomarkers, we performed differential abundance analyses using both LEfSe and the Kruskal-Wallis test followed by Dunn’s test with Holm’s correction. All four group comparisons with LEfSe identified *g_Clostridium_sensu_stricto_1* (LDA score = 4.13; *p* < 0.01), *g_Romboutsia* (LDA score = 4.07; *p* < 0.01), and *f_Peptostreptococcaceae* (LDA score = 4.13; *p* < 0.01) as distinguishing features for PD group. For the UKT2D group, an unknown species in the *Veillonella* genus (LDA score = 3.04; *p* < 0.01), *f_Atopobiaceae* (LDA score = 3.80; *p* < 0.01), and *g_Fournierella* (LDA score = 4.35; *p* < 0.01) were potential distinguishers. In the Ctrl group, *g_Terrisporobacter* (LDA score = 3.64; *p* = 0.01) and an unknown species in taxa *Gastranaerophilales* (LDA score = 3.15; *p* < 0.04) were distinguishing. Notably, no features were identified for KT2D in the conducted analysis (Figure S1A). Pairwise comparisons at all taxonomy levels further delineated group differences. *s_Bifidobacterium angulatum* (LDA score = 4.17; *p* < 0.03) was differentially abundant in the Ctrl group compared to UKT2D (Figure S1B). *g_Akkermansia* (LDA score = 3.87; *p* < 0.05) was the differentiator between the Ctrl and KT2D (Figure S1C). Although no taxa associated with PD group showed significantly higher abundance compared to UKT2D group, several taxa, including *g_Collinsella* (LDA score = 3.41; *p* = 0.04) and *s_Lactobacillus porci* (LDA score = 3.53; *p* < 0.01), were more abundant in the latter (Figure S1D). In comparisons between the Ctrl and KT2D, *g_Akkermansia* (LDA score = 3.87; *p* < 0.05) was differentially abundant in KT2D (Figure S1E). Only the UKT2D group had several taxa, including *g_prevotella* (LDA score = 4.50; *p* < 0.02), *g_Faecalibacterium* (LDA score = 4.04; *p* < 0.01), *Streptococcus salivarius* (LDA score = 3.78; *p* = 0.02), and *Streptococcus anginosus* (LDA score = 3.00; *p* < 0.03) differentially abundant in comparison with KT2D (Figure S1F). Post-hoc Dunn’s test with Holm correction revealed significant changes. *Fournierella* (df, Obs. = 4%, KT2D = 3%, UKT2D = 20%, PD = 0%, Ctrl = 0%) altered in all comparisons involving UKT2D; *Romboutsia* (df, Obs. = 48%, KT2D = 18%, UKT2D = 67%, PD = 76%, Ctrl = 67%) showed changes in comparisons with KT2D. *Barnesiella* (df, Obs. = 12%, KT2D = 5%, UKT2D = 33%, PD = 8%, Ctrl = 13%) had significance between UKT2D and KT2D (*p* = 0.036), *Clostridium_sensu_stricto_1* was differentially abundant between the Ctrl and KT2D (*p* = 0.009) and PD with KT2D (*p* = 0.0002). *Coriobacteriaceae_UCG-003* (df, Obs. = 24%, KT2D = 10%, UKT2D = 47%, PD = 32%, Ctrl = 27%) was differentially abundant between UKT2D and KT2D (*p* = 0.013), as was *Faecalibacterium* (df, Obs. = 86%, KT2D = 75%, UKT2D = 100%, PD = 92%, Ctrl = 93%; *p* = 0.018). However, *Prevotella* (df, Obs. = 2%, KT2D = 0%, UKT2D = 0%, PD = 8%, Ctrl = 0%) and *Veillonella* (df, Obs. = 17%, KT2D = 5%, UKT2D = 33%, PD = 28%, Ctrl = 13%) lost significance at a 95% CI.

Altogether, these differential abundance analyses using both LEfSe and Dunn’s test revealed distinct microbial signatures across the experimental cohorts. Shared features, such as *Fournierella*, *Rombutsia*, and *Clostridium*_*sensu_stricto_1*, found in both UKT2D and KT2D, highlighting the gut microbiome alterations associated with different stages of diabetes progression. Although certain taxa like *Prevotella* and *Veillonella* lost their significance at a 95% CI in Dunn’s test, they were identified as potential biomarkers in LEfSe analysis.

### Distinct correlation arrays reveal specific microbiome signatures linked with clinical parameters relevant to T2D

**Figure 3:**
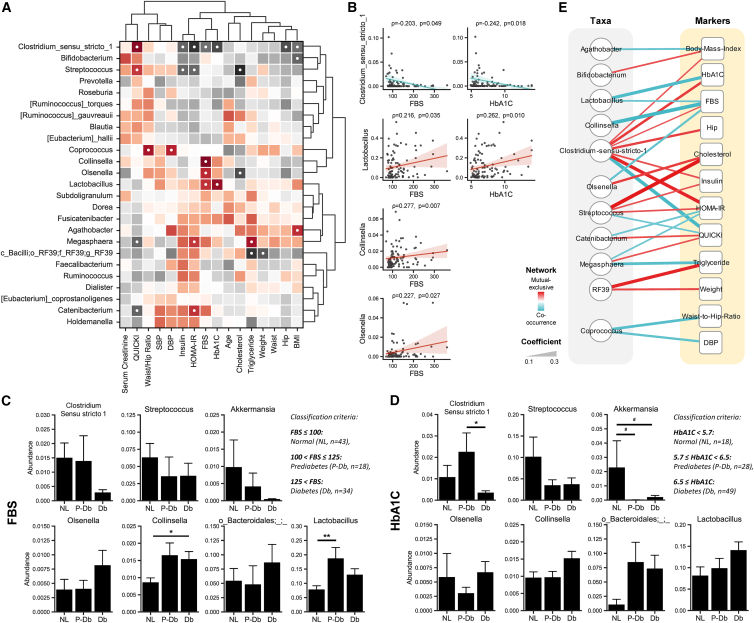
Distinct correlation arrays reveal specific microbiome signatures linked with clinical parameters relevant to T2D (A) Spearman’s rank correlation analysis of bacterial genera with a prevalence of >50% and with significant relationships (*p* < 0.05) marked by “●.” (B) Linear regression plot showing the direction of the correlation of taxa specifically with HbA1c and FBS. (C) Co-occurrence network of biochemical and anthropometric markers constructed using Spearman’s rank correlation results. Circular nodes represent bacterial taxa and square nodes represent clinical markers. Edges represent significant correlations (*p* < 0.05) and are weighted according to correlation strength. (D–E) Variation in taxa abundance based on the classification of subjects: (D) By FBS (classification criteria: FBS ≤100: normal [NL, *n* = 43], 100 < FBS ≤125: prediabetes [P-Db, *n* = 18], FBS >125, diabetes [Db, *n* = 34]). (E) By HbA1c (classification criteria: HbA1c < 5.7: normal [NL, *n* = 18], 5.7 ≤ HbA1c < 6.5: prediabetes [P-Db, *n* = 28], HbA1c ≥ 6.5: diabetes [Db, *n* = 49]). #*p* < 0.1; ∗*p* < 0.05; ∗∗*p* < 0.01.

To assess the link between gut microbiome and clinical parameters of T2D, we conducted correlation analyses on 25 prevalent bacterial genera (prevalence >50%) (Figure 3A). *Clostridium_sensu_stricto_1* was negatively correlated with both HbA1c (R = −0.24, *p* = 0.01) and FBS (R = −0.20, *p* = 0.04), while *Lactobacillus* (R = 0.26/0.21, *p* = 0.01/0.03) exhibited positive correlations with these parameters. *Collinsella* (df, Obs. = 94%, KT2D = 93%, UKT2D = 100%, PD = 88%, Ctrl = 100%) and *Olsenella* (df, Obs. = 63%, KT2D = 55%, UKT2D = 80%, PD = 56%, Ctrl = 80%) were also positively associated with FBS (R = 0.27/0.22, *p* < 0.01/ = 0.02). Furthermore, *Streptococcus* and *Clostridium_sensu_stricto_1* showed positive associations with the QUICKI (r = 0.21/0.27, *p* = 0.03/<0.01) and negative associations with the HOMA-IR (R = −0.21/-0.27, *p* = 0.03/<0.01). Conversely, *Megasphaera* (df, Obs. = 53%, KT2D = 53%, UKT2D = 67%, PD = 48%, Ctrl = 47%) and *Catenibacterium* (df, Obs. = 78%, KT2D = 68%, UKT2D = 80%, PD = 84%, Ctrl = 93%) displayed opposite trends (QUICKI, r = −0.21/-0.21, *p* = 0.03/0.03; HOMA-IR, r = 0.21/0.21, *p* = 0.03/0.03). Additionally, we observed a negative correlation between *Clostridium_sensu_stricto_1* with BMI (r = −0.20, *p* = 0.04) and hip circumference (r = −0.23, *p* = 0.02). To validate these findings and investigate the abundance of specific microbial genera relative to FBS and HbA1c levels, a Spearman’s rank correlation and one-way ANOVA with Tukey-HSD post-hoc analysis were performed (Figures 3A–3E). Based on FBS level, subjects were classified into three groups, normal (NL, ≤100 mg/dL; *n* = 43), prediabetic (P-Db, 100 < FBS ≤125 mg/dL; *n* = 18), and diabetic (Db, >125 mg/dL; *n* = 34). For HbA1c, subjects were classified as normal (NL, <5.7%; *n* = 18), prediabetic (P-Db, 5.7 ≤ HbA1c < 6.5%; *n* = 28), and diabetic (Db, ≥6.5%; *n* = 49). *Clostridium_sensu_stricto_1* significantly correlated with both FBS (*p* = 0.049) and HbA1c (*p* = 0.018) (Figure 3B). For the FBS, *Clostridium_sensu_stricto_1* was more abundant in the NL and P-Db groups compared to Db, although these differences were not statistically significant (Figure 3C). For HbA1c, *Clostridium_sensu_stricto_1* was most abundant in P-Db, with significant differences observed between P-Db and Db (*p* < 0.05) (Figure 3D). *Collinsella* showed significantly lower abundance in NL compared to P-Db and Db (*p* < 0.05) and demonstrated a positive correlational relationship (*p* = 0.007) with FBS (Figures 3A, 3B, and 3D). *Lactobacillus* was significantly less abundant in NL compared to P-Db (*p* < 0.01), with intermediate abundance in Db for FBS, and showed a positive correlation with both FBS and HbA1c (Figures 3A–3C). *Akkermansia* was most abundant in NL, least abundant in P-Db, and showed intermediate abundance in Db, with both comparisons being significant at a 90% CI for HbA1c (Figure 3D). Furthermore, we conducted network analysis to explore the co-occurrence of clinical parameters and microbial taxa, filtering correlations with coefficients >0.2 at a 95% CI (Figure 3E). *Clostridium_sensu_stricto_1* had the highest number of connections, being significantly positively correlated with BMI, HbA1c, FBS, hip circumference, insulin, and HOMA-IR, but negatively correlated with QUICKI. Genera with single significant connection included *Agathobacter* (df, Obs. = 84%, KT2D = 80%, UKT2D = 93%, PD = 88%, Ctrl = 80%) and *Bifidobacterium*, both associated with BMI, and *Collinsella*, which was strongly negatively connected to FBS.

These results highlight the complex interactions between gut microbiota and clinical parameters in T2D. Notably, the findings also indicate that specific bacterial taxa, such as *Clostridium_sensu_stricto_1*, could prove to be potential biomarkers and could also be exploited as therapeutic targets due to their significant correlations with key clinical metrics of glucose metabolism and adiposity.

### Metformin modulates the gut microbiome in insulin-resistant subjects without improving insulin sensitivity

**Figure 4:**
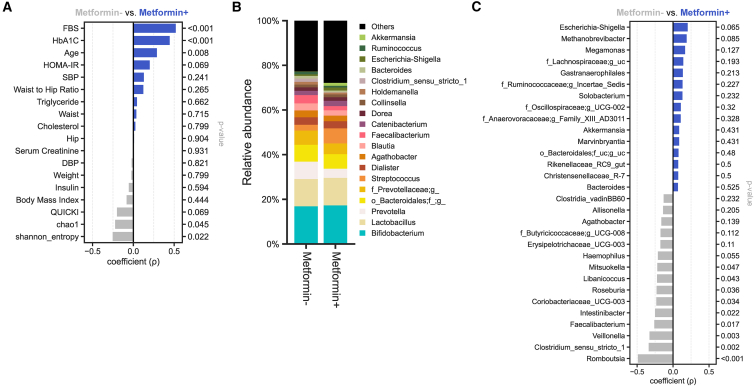
Differences in gut microbiome and T2D-relevant clinical biomarkers based on Metformin administration (A) Spearman’s rank correlation analysis between Metformin users (Metformin+) and non-users (Metformin–) for anthropometric and biochemical markers. (B) Genus-level microbial composition in Metformin+ and Metformin– subjects. (C) Spearman’s rank correlation analysis between Metformin+ and Metformin– subjects for bacterial genera.

We subsequently investigated the impact of metformin, a commonly prescribed biguanide for T2D, on gut microbiome and metabolic parameters in insulin-resistant individuals. Considering the insulin resistance in PD subjects, we categorized them in the metformin-group. Consequentially, significant alterations in HbA1c and FBS levels were displayed, even though there was no observed change in insulin sensitivity. Hence, it is crucial to interpret these findings cautiously. However, no significant changes were observed in insulin resistance and sensitivity parameters, namely HOMA-IR and QUICKI, within the 95% CI. Additionally, the metformin-treated (Metformin+) group had a significantly higher mean age compared to the non-metformin-treated (Metformin-) group (*p* = 0.008). Diversity measures of the gut microbiome indicated that the Shannon (*p* = 0.022) diversity was significantly higher in the Metformin-group (Figure 4A). The comparative analysis of gut microbiomes between Metformin+ and Metformin-subjects revealed a significant reduction in several taxa within the Metformin+ group, including *Mitsuokella* (*p* = 0.047), *Libanicoccus* (*p* = 0.043), *Roseburia* (*p* = 0.036), *Coriobacteriaceae_UCG_003* (*p* = 0.034), *Intestinibacter* (*p* = 0.022), *Faecalibacterium* (*p* = 0.017), *Veillonella* (*p* = 0.003), *Clostridium_sensu_stricto_1* (*p* = 0.002), and *Rombutsia* (*p* < 0.001). No significant increases in bacterial taxa were observed in the Metformin+ group within the 95% CI (Figures 4B and 4C).

These results demonstrate that metformin use is associated with notable differences in the gut microbiome composition between insulin-resistant subjects, with and without metformin treatment. However, these microbiome alterations didn’t correspond with improvements in insulin sensitivity. Additionally, the lower microbial diversity, as measured by the Shannon entropy index in Metformin+ subjects, suggests that while metformin influences the gut microbiome, these changes may not be linked with insulin sensitivity.

### Alterations in lipid metabolism, xenobiotic degradation, and signal transduction pathways are predicted in cohorts with different T2D progression stage

**Figure 5:**
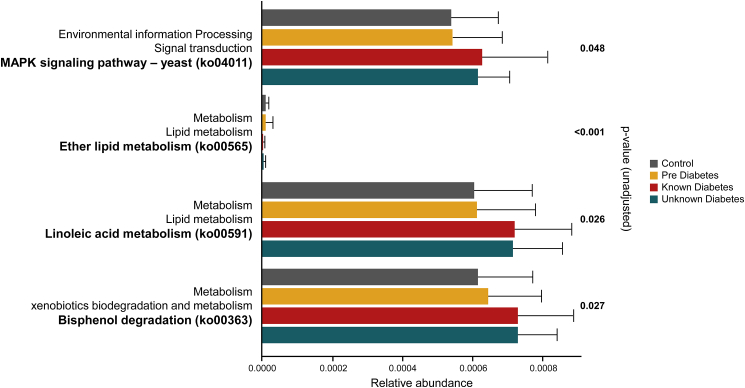
Functional pathway prediction. Alterations in the microbiome-associated metabolic activity arrays in subjects belonging to different experimental groups, as predicted using PICRUSt2 followed by ALDEx2

Metabolic pathways identified as altered among the groups of KT2D, UKT2D, PD, and Ctrl using the ALDEx2 tool included the MAPK signaling pathway, ether lipid metabolism, linoleic acid metabolism, and bisphenol degradation. The gut microbiome of the KT2D and UKT2D groups exhibited increased activity in three out of the four pathways compared to the PD and Ctrl (Figure 5). The only metabolic pathway with reduced activity in the KT2D and UKT2D groups was ether lipid metabolism. Bisphenol degradation was more active in the PD group relative to the Ctrl group, while the other altered pathways showed comparable activity levels between the PD and Ctrl groups. The similar peak activity observed in KT2D and UKT2D across the altered pathways suggests analogous functional changes under different disease states or similar environmental exposures.

## Discussion

This is among the first studies examining the gut microbiome profiles, by using high-throughput long-read V1-V9 sequencing approach, in an Indian clinical cohort involving subjects at different stages of T2DM progression and medication regimen. The findings clearly delineate significant divergence in the gut microbiome profiles at various stages of T2D, particularly noting distinct microbial patterns in KT2D, UKT2D, and PD compared to Ctrl subjects. This highlights the complex role of the gut microbiome in T2D progression and its potential therapeutic implications.

The genus *Akkermansia*, a well-recognized feature in gut microbiome studies related to T2D, showed intriguing but contrasting results. High levels of *Akkermansia* were observed in few samples, skewing the mean values. Notably, metformin users exhibiting an elevated abundance of *Akkermansia* (though this finding should be considered with caution due to its uneven distribution, prevalence, and abundance) tended to have HbA1c levels closer to those of the Ctrl group. This pattern aligns with existing literature, although associations were stronger with BMI than HbA1c in our study population.^7^^,^^15^ This discrepancy might be due to the distinct interactions of T2D and BMI in south Asian populations compared to Western counterparts, where BMI is more strongly correlated with T2D.^16^ We observed that anti-diabetic medications might selectively promote certain bacterial genera such as *Akkermansia*,^7^^,^^8^^,^^17^^,^^18^^,^^19^ yet may reduce overall microbiome diversity.^12^^,^^20^ However, the mechanisms by which these drugs affect the microbiome remain unclear and warrant further investigation.

Notably, no significant differences were observed in HbA1c, QUICKI, and HOMA-IR levels between UKT2D and KT2D groups, raising questions about the extent of glycemic improvement associated with current treatment regimens in this population. Similar issues with the efficacy of these medications have been reported in other Asian studies.^21^ Additionally, a meta-analysis found only modest improvements in HbA1c levels in individuals already on medication compared to those who started treatment within the first 4 to 6 months.^22^ Our data indicates a significant microbiome deviation in PD compared to other groups, and diminished gut diversity in KT2D. This contrasts with existing findings from studies on the Indian population but aligns with observations from studies on Arab and US populations.^23^^,^^24^^,^^25^

We identified a significant abundance of *Clostridium_sensu_stricto_1* in PD individuals, negatively correlated with HbA1c and FBS, indicating a potential role in glucose metabolism. This genus was also positively correlated with QUICKI, suggesting an intricate involvement in glucose homeostasis. This genus was more abundant in the NL and P-Db groups compared to the Db group (classified based on HbA1c and FBS criteria), with statistical significance observed only between P-Db and Db for HbA1c. These results align with previous studies^5^^,^^6^ linking higher abundance of *Clostridium_sensu_stricto_1*, a butyrate-producing bacterium known for maintaining gut barrier integrity, with lower incidence of T2D.^26^^,^^27^ In our study, network analysis further revealed significant correlations of aforementioned genus with BMI, HbA1c, FBS, hip circumference, insulin, HOMA-IR, and QUICKI, underscoring its potential importance in glucose homeostasis and insulin sensitivity within our population.

*Lactobacillus* showed positive correlations with HbA1c and FBS, while *Collinsella* and *Olsenella* were also positively associated with FBS, suggesting that these genera may contribute to blood sugar levels homeostasis in T2D or thrive due to changes in the gut environment. The findings for *Lactobacillus* align with previous research,^28^^,^^29^^,^^30^ indicating that its increased abundance correlates with higher blood glucose levels, likely due to the ample availability of simple sugars. However, it does not directly metabolize lactate; thus, excessive lactate produced by *Lactobacillus* may enter the liver and be converted to glucose, ultimately re-entering the bloodstream. Thus, its impact on glucose metabolism may be limited by its reliance on hepatic gluconeogenesis.^31^^,^^32^^,^^33^ Additionally, *Veillonella*, which produces SCFAs by fermenting lactate and aids in blood glucose homeostasis, had a lower df but has been used as a biomarker to differentiate experimental groups.^34^ On the other hand, *Megasphaera*, a simple sugar fermenter considered a hallmark in the Indian gut, and *Catenibacterium*, which ferments carbohydrates to produce acetic acid (a metabolite known to improve insulin sensitivity), showed positive associations with HOMA-IR.^35^^,^^36^ However, the abundance of *Catenibacterium* was the lowest in PD patients (df, Obs. = 78%, KT2D = 68%, UKT2D = 80%, PD = 84%, Ctrl = 93%). Such associations of these metabolically active genera with HOMA-IR suggest context-dependent effects of gut microbes on insulin sensitivity. Further functional interrogations using PICRUSt2 highlighted an increased activity of the Bisphenol-A degradation pathway and the MAPK signaling pathway in diabetics, suggesting heightened exposure to Bisphenol-A and possible compromised gut barrier integrity, respectively. However, a lack of detailed diet logs limits our ability to confirm these findings.

Our findings reveal distinct gut microbiome patterns associated with T2D progression and medication status in a south Asian (Indian) population, highlighting potential microbial markers such as *Clostridium_sensu_stricto_1*, which may be involved in metabolic regulation. Notably, key glycemic, insulin sensitivity, and insulin resistance markers were largely comparable between treated and untreated T2D groups, despite pronounced differences in gut microbiome composition. In addition, lactate-metabolizing genera such as *Veillonella* may have a potential role in modulating glycemic homeostasis. While the cross-sectional nature of this study precludes inference on the microbiome’s role in T2D onset, the observed associations support existing evidence linking gut microbiome alterations with disease progression. Together, these findings underscore the need for future longitudinal and mechanistic studies across diverse populations to clarify host-microbe interactions and to evaluate the therapeutic potential of gut microbiome modulation in T2D management.

### Limitations of the study

Interpretation of these findings was constrained by age disparities between groups, cross-sectional design, and the lack of larger, evenly distributed samples restricting evaluation and generalizability anti-diabetic drug efficacy in south Asian population. Our amplicon sequencing approach also limits insights into other non-bacterial gut microbial residents, such as fungi and viruses, necessitating future research using whole community metagenomic or transcriptomic techniques. Additionally, although we observed associations between specific taxa such as *Clostridium sensu stricto_1* and *Lactobacillus* with glycemic parameters, these analyses were limited to cross-sectional correlations. While multivariate regression adjusting for age, sex, and BMI intake was performed (Data S2), functional validation of microbial genes and causal inference through animal studies were not feasible in this observational study and will be pursued in future investigations. Furthermore, while metformin was associated with shifts in the gut microbiota, the absence of pre-treatment baseline samples and the imbalance between metformin users and non-users limited our ability to establish causal drug, microbiota, and T2D relationships.

## Resource availability

### Lead contact

Further information and requests for resources should be directed to and will be fulfilled by the lead contact, Manoj Kumar (manoj15ndri@gmail.com).

### Materials availability

This study did not generate new reagents. Any material used in the study is available from the authors upon reasonable request.

### Data and code availability

The datasets generated and analyzed in this study are provided herein as supplemental information. The raw sequences supporting the findings of this study are openly available in National Center for Biotechnology Information (NCBI)-Sequence Read Archive (SRA) at https://www.ncbi.nlm.nih.gov/sra, under reference number BioProject: PRJNA1224288. The code used for the analyses in this study is available at the GitHub repository: https://github.com/Raj-Analysis/ut2d-t2d-microbiome.

## Acknowledgments

This study was funded by a grant from the Indian Council of Medical Research (ICMR) grant no. GIA/2019/000221/PRCGIA to M.K. The authors wish to thank fellow colleagues, clinical staff members, nurses, students, and study participants that helped and cooperated in this study.

## Author contributions

M. Kumar, S. Singh, R.O., R.N., and V.V. contributed equally to the conceptualization and execution of this study. M. Kumar secured funding for the study. R.N., G.P., R.O., and M. Kumar analyzed and interpreted the data. M. Kriti, P.S., and N.P. accessed and verified the underlying data before manuscript submission. M. Kumar, S. Singh, R.O., R.N., and V.V. drafted the manuscript. G.P. and R.O. pre-processed the imaging data. S. Shubham, M.K.P., M. Kumar, R.R.T., and D.J.P. contributed to editing the manuscript. All authors approved the final version.

## Declaration of interests

The authors declare no competing financial interests or personal relationships that could have influenced the work presented in this study.

## STAR★Methods

### Key resources table

**Table:** 

REAGENT or RESOURCE	SOURCE	IDENTIFIER
**Biological samples**		

Fecal	Human Participant	N/A
Whole Blood	Human Participant	N/A

**Critical commercial assays**		

DNeasy PowerSoil Pro Kit	Qiagen	Cat#47016
DNA HS Assay Kit	Invitrogen	Cat#Q32851
KAPA HiFi HotStart ReadyMix	Roche	Cat#KK2602
Express Template Preparation Kit	Pacific Biosciences	PN#100-938-900
Sequel II Binding Kit 2.1	Pacific Biosciences	PN#101-820-500
Triglyceride Test Kit	Erba	Cat#BLT00057
Cholesterol Test Kit	Erba	Cat#XSYS0009
Creatinine Test Kit	Erba	Cat#BLT00020
Ultrasensitive Insulin Test	Beckman Coulter	Cat#BC33410
HbA1c 501 Test Cartridge	HemoCue	REF#405116
Glucose 201 Microcuvettes	HemoCue	PN#BCH553

**Deposited data**		

16S rRNA (V1-V9) amplicon sequencing data	NCBI SRA	PRJNA1224288

**Oligonucleotides**		

Barcoded 16S Primers	Pacific Biosciences	N/A

**Software and algorithms**		

RStudio	Posit Software	https://posit.co/downloads/
R	R Foundation for Statistical Computing	https://www.r-project.org/
Python	Python Software Foundation	https://www.python.org/
Cytoscape (V3.10.2)	Cytoscape Consortium	https://cytoscape.org/
QIIME2 (V2-2023.5)	QIIME2 Development team	https://qiime2.org/
InteractiVenn	InteractiVenn: a web-based tool for the analysis of sets through Venn diagrams (https://doi.org/10.1186/s12859-015-0611-3)	http://www.interactivenn.net/citation.html

### Experimental model and study participant details

#### Study population

The study was approved by the Institutional Ethics Committee (IEC) of ICMR–NIREH (Approval No. NIREH/BPL/IEC/2020-21/43), and all procedures were conducted in accordance with relevant guidelines and regulations. The sample collection drives were conducted in Bhopal, Madhya Pradesh, India, surveying more than 500 households. Only individuals aged 18 to 65, free from severe health conditions, were considered for the study. From this, 95 individuals (Female *n* = 48, Male *n* = 47) with a mean age of 49 ± 9.34, voluntarily provided the requisite samples, i.e., blood and feces, after providing written informed consent. Subjects were meticulously classified based on their pre-diagnosed status/declaration of T2D. Those already with a clinical diagnosis of T2D and were on medications for the same, regardless of treatment duration, were enrolled under the KT2D category (*n* = 40). As accurate and consistent data on duration were not consistently available among participants, it was dropped from analysis. Subjects having glycated hemoglobin (HbA1c) levels in the diabetic range (i.e., ≥6.5% DCCT units), who had not been previously diagnosed clinically for T2D and were not on anti-diabetic medications, were classified under UKT2D (*n* = 15). The remaining subjects were classified as per the HbA1c cut-off ranges provided by the American Diabetes Association (ADA); PD (DCCT units 5.7%–6.4%; *n* = 25) and normoglycemic Ctrl (DCCT units <5.7%; *n* = 15).

### Method details

#### Clinical parameters

Serum was collected by centrifuging the blood collected in plain glass/gel vial after allowing it to clot. The clinical analytes including serum creatinine, triglycerides, cholesterol, and fasting Insulin were measured. The HbA1c and fasting blood sugar (FBS) readings were taken in the field with finger-pricking method from overnight-fasted subjects. Anthropometric measurements were taken for height, weight, waist, and hip. Based on these, the Body Mass Index (BMI), waist-to-hip ratio (WHR), and waist-to-height ratio (WHtR) were calculated. Additionally, indices such as Homeostasis Model Assessment of β-cell and Insulin Resistance (HOMA-B/-IR) and Quantitative Insulin Sensitivity Check Index (QUICKI) were calculated (Table S1).

#### Sample preparation and sequencing

The freshly collected fecal samples were stored at −80C in the freezing facility of the institute and were processed for high-quality metagenomic DNA isolation within a median of 3 days. The DNA isolation was performed using QIAGEN DNeasy PowerSoil Pro Kit with the manufacturer’s recommended protocol. After checking for quality and quantity of the isolated DNA samples, PCR amplification was performed using universal primers for full-length 16S rRNA V1-V9 region (PacBio). Library preparation was done with Express Template Preparation kit 2.0 with an initial input quantity of 1.7 μg of amplified PCR products. Reaction composition, thermal cycling conditions, and other library preparation parameters are provided in Tables S2–S4. The final Single Molecule Real Time (SMRT) library size obtained was 1903 bp. The binding kit used was Sequel II Binding kit 2.1, and 90 pM library was loaded onto the SMRT cell of PacBio Sequel II sequencing platform, containing 8M zero mode wave guides. This generated, on average, 872,446 subreads with an overall average length of 1523.78 bp for this project.

### Quantification and statistical analysis

The overall bioinformatics analysis was performed as per our previously described methods.^37^^,^^38^^,^^39^ Briefly, the microbiome sequencing data was processed using QIIME2 (version 2–2023.5).^40^ The raw sequences underwent demultiplexing and quality filtering with the q2-demux plugin. This was followed by dereplication, denoising, and chimera filtering using DADA2-CCS, which is optimized for single-end PacBio CCS sequences.^41^ All reads were processed using the default parameters: reads were required to contain the forward primer (allowing up to two mismatches), primers were removed automatically, no defined-length trimming was applied, quality filtering was based on a maximum expected error threshold of 2.0, denoising was conducted independently for each sample, and consensus-based chimera removal was performed, following standard QIIME 2 recommendations. The identified amplicon sequence variants (ASVs) were aligned with MAFFT.^42^ Taxonomy assignment of the ASVs was performed using the sklearn classifier with a pre-trained naive Bayes taxonomy classifier, aligned against the 99% SILVA 138 database. In addition, rarefaction was applied for normalization prior to calculating microbial diversity. The rarefaction depth was set to 11,322, and the program randomly subsampled, each sample to this sequencing depth. All alpha and beta diversity metrics were then calculated using the rarefied feature table. Alpha diversity was measured using observed ASVs (richness) and the Shannon index (richness and evenness). For beta diversity, the Bray-Curtis dissimilarity index was employed and visualized through principal coordinate analysis (PCoA). Significant differences in microbial diversity and structure were identified using the non-parametric Kruskal-Wallis test and PERMANOVA with 999 random permutations. Differential abundance of taxa between groups was identified using Linear Discriminant Analysis (LDA) effect size (LEfSe) and ANOVA-Like Differential Expression (ALDEx2).^43^ Spearman’s correlation was used to assess the association between taxa and physiological markers, and to identify taxa more abundant based on HbA1c classification or Metformin consumption. A two-way ANOVA was conducted to evaluate the impact of age and sex on taxa abundance or physiological markers and their interaction effects. Networks linking taxa and physiological markers were constructed by calculating Spearman correlations, and significant associations (Spearman correlation coefficient > 0.20 and Benjamini–Hochberg corrected *p* < 0.05) were visualized using Cytoscape v3.10.2.^44^ Comparisons involving more than three groups were conducted with a one-way ANOVA test followed by Tukey-HSD post-hoc analysis. Venn diagrams were illustrated using InteractiVenn.^45^ Metagenomic functional profiling was performed using PICRUSt2 (Phylogenetic Investigation of Communities by Reconstruction of Unobserved States 2), an open-source bioinformatics tool. Input sequences were used to predict the functional gene content of taxonomically classified gut microbiota. The inferred gene families were annotated with KEGG (Kyoto Encyclopedia of Genes and Genomes) orthologs and organized into KEGG pathways to characterize functional profiles. Differentially abundant predicted pathways were identified based on ALDEx2 analysis. Visualization was performed using ‘R’ or ‘Python’ packages. De-identified subject-level metadata and genus level normalised taxonomic abundance table (top 50) derived from these samples are provided in Data S1.

## Supplemental information

Document S1. Figure S1 and Tables S1–S4

Data S1. De-identified participant metadata and genus level normalized abundance matrix (top 50 genera)

Data S2. Multivariate regression results adjusted for age, sex, and BMI

Data S3. (A–G) LEfSe results. (A) All four group; (B) Ctrl vs. KT2D; (C) Ctrl vs. PD; (D) Ctrl vs. UKT2D; (E) KT2D vs. UKT2D; (B) PD vs. KT2D; (G) PD vs. KT2D
