# Supplementary material for: Gut microbiome disparities reflect type 2 diabetes progression and medication status
Source: iScience. 2026 Feb 26;29(3):115156. doi: 10.1016/j.isci.2026.115156 (PMC12997337; doi:10.1016/j.isci.2026.115156)
Supplement: Document S1. Figure S1 and Tables S1–S4 [file mmc1.pdf]

iScience, Volume *29*

Supplemental information

Gut microbiome disparities reflect type 2 diabetes progression and medication status

Manoj Kumar, Samradhi Singh, Raj Ojha, Mona Kriti, Gwoncheol Park, Vinod Verma, Namrata Pal, Poonam Sharma, Swasti Shubham, Megha K. Pandey, Devraj J. Parasannanavar, Devojit K. Sarma, Rajnarayan R. Tiwari, and Ravinder Nagpal

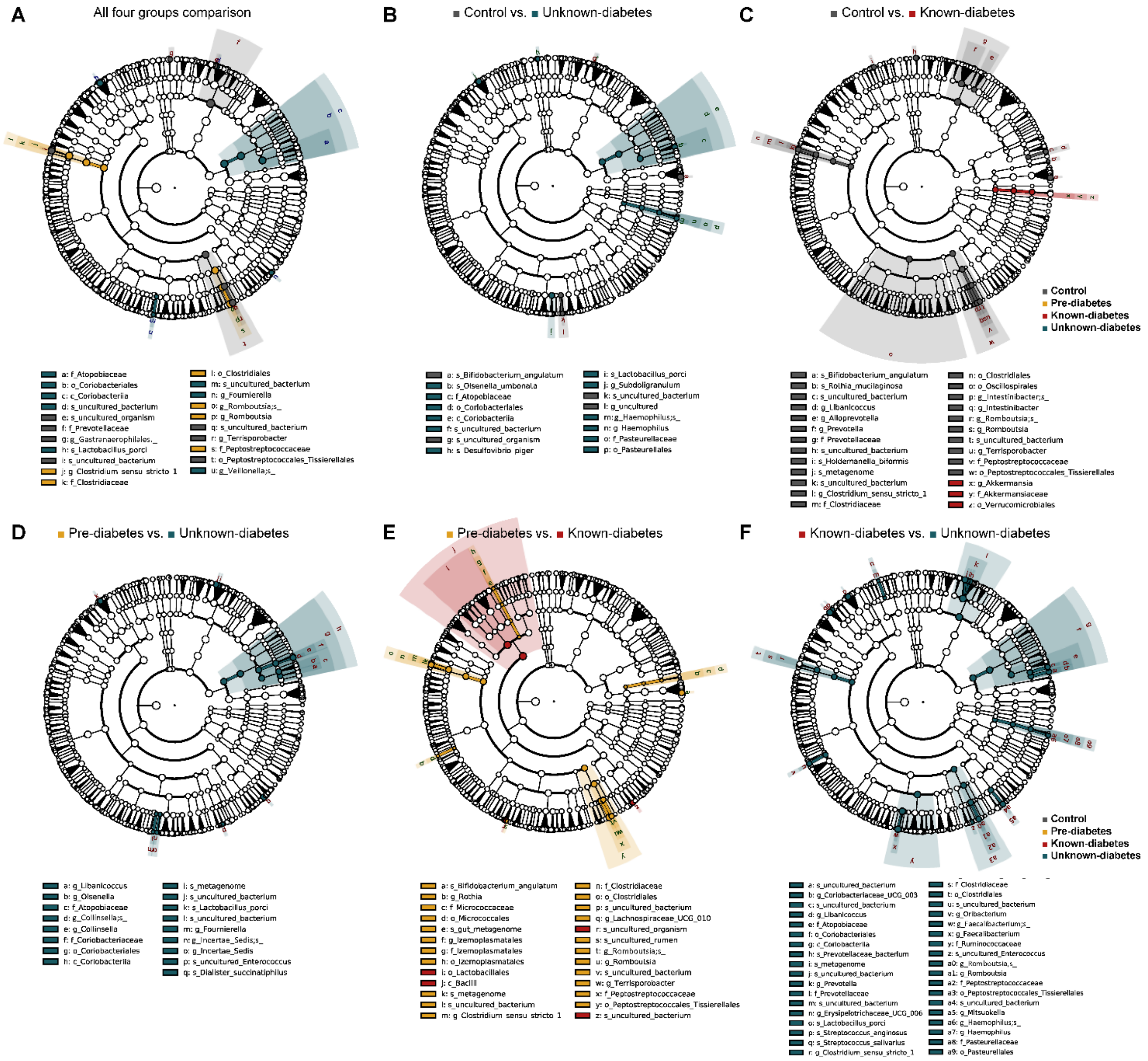

Figure S1. Gut microbiome disparities in subjects with different stage of T2D progression.

(A-F) Cladograms generated using Linear Discriminant Analysis Effect Size (LEfSe) to identify taxa differentially abundant between groups. Differential features were determined using the Kruskal–Wallis test followed by pairwise Wilcoxon tests ($p < 0.05$). **(A)** All four groups; **(B)** Ctrl vs UKT2D; **(C)** Ctrl vs KT2D; **(D)** PD vs UKT2D; **(E)** PD vs KT2D; **(F)** KT2D vs UKT2D.

Index	Overall			KT2DM			UKT2DM			PD			Ctrl		
	**M**	**F**	**n**	**M**	**F**	**n**	**M**	**F**	**n**	**M**	**F**	**n**	**M**	**F**	**n**
Count	47	48	95	18	22	40	9	6	15	13	12	25	7	8	15
Age	50.79 (9.66)	48.63 (8.98)	49.69 (9.34)	54.39 (10.04)	53.86 (7.08)	54.10 (8.43)	49.00 (10.05)	44.33 (7.09)	47.13 (9.01)	50.23 (8.07)	44.83 (7.49)	47.64 (8.12)	44.86 (8.91)	43.13 (10.27)	43.93 (9.36)
SBP	137.34 (17.72)	134.38 (17.78)	135.84 (17.72)	138.39 (16.40)	138.64 (18.37)	138.52 (17.28)	141.78 (15.59)	140.67 (27.21)	141.33 (20.09)	129.85 (14.37)	131.00 (10.22)	130.40 (12.31)	142.86 (26.78)	123.00 (12.64)	132.27 (22.19)
DBP	87.85 (11.32)	80.73 (10.40)	84.25 (11.39)	86.00 (10.46)	81.14 (10.96)	83.32 (10.88)	93.00 (10.23)	83.57 (12.31)	89.20 (11.71)	85.54 (12.58)	82.08 (8.28)	83.88 (10.66)	90.29 (12.18)	75.50 (10.45)	82.40 (13.28)
FBS	130.23 (62.04)	126.88 (51.51)	128.54 (56.69)	157.22 (66.20)	163.45 (55.95)	160.65 (60.04)	163.44 (76.59)	106.83 (14.99)	140.80 (65.24)	90.62 (11.00)	94.42 (13.41)	92.44 (12.11)	91.71 (7.74)	90.00 (8.60)	90.80 (7.97)
HbA1c	7.18 (2.10)	7.23 (2.04)	7.20 (2.06)	8.07 (1.75)	8.68 (2.20)	8.40 (2.01)	8.72 (2.91)	6.83 (0.31)	7.97 (2.40)	5.88 (0.19)	5.97 (0.19)	5.92 (0.19)	5.30 (0.23)	5.40 (0.12)	5.35 (0.18)
Cholesterol	154.17 (46.70)	166.64 (59.61)	160.47 (53.69)	149.08 (42.84)	181.02 (78.07)	166.65 (65.88)	155.92 (31.85)	155.72 (38.67)	155.84 (33.37)	180.57 (54.49)	148.74 (39.45)	165.29 (49.61)	115.96 (30.54)	162.14 (27.09)	140.59 (36.54)
Triglyceride	116.04 (106.26)	77.88 (78.23)	96.76 (94.61)	127.14 (135.31)	103.71 (92.15)	114.25 (112.66)	98.93 (58.68)	45.35 (21.55)	77.50 (53.59)	138.23 (107.46)	58.97 (57.71)	100.19 (94.52)	68.27 (49.66)	59.64 (77.77)	63.67 (64.04)
S. Crt.*	0.90 (0.22)	0.77 (0.16)	0.83 (0.20)	0.91 (0.12)	0.76 (0.21)	0.82 (0.19)	0.92 (0.12)	0.73 (0.11)	0.85 (0.15)	0.86 (0.38)	0.79 (0.08)	0.82 (0.28)	0.90 (0.18)	0.79 (0.11)	0.84 (0.15)
Insulin	7.14 (6.47)	9.78 (5.66)	8.47 (6.18)	8.28 (8.27)	9.55 (5.57)	8.97 (6.85)	7.64 (7.87)	9.80 (4.94)	8.51 (6.73)	6.80 (3.48)	12.14 (6.03)	9.36 (5.49)	4.24 (2.35)	6.84 (5.24)	5.62 (4.23)
Height	168.34 (6.68)	153.27 (7.79)	160.72 (10.46)	169.89 (6.13)	153.41 (7.44)	160.82 (10.72)	165.89 (3.69)	149.83 (5.98)	159.47 (9.32)	169.62 (5.55)	154.25 (7.64)	162.24 (10.17)	165.14 (11.14)	154.00 (10.56)	159.20 (11.92)
Weight	70.89 (12.49)	65.15 (12.65)	67.99 (12.83)	72.27 (10.32)	66.46 (12.81)	69.07 (11.97)	73.40 (11.93)	60.82 (10.84)	68.37 (12.81)	73.58 (14.06)	68.83 (11.69)	71.30 (12.94)	59.14 (10.79)	59.29 (14.12)	59.22 (12.23)
Waist	95.33 (8.13)	94.76 (11.71)	95.04 (10.04)	96.75 (8.31)	97.25 (11.58)	97.02 (10.11)	98.88 (6.46)	89.62 (12.31)	95.17 (10.00)	94.94 (7.06)	95.33 (11.61)	95.13 (9.31)	87.86 (8.11)	90.90 (11.75)	89.48 (9.99)
Hip	96.54 (7.92)	102.40 (10.23)	99.50 (9.57)	96.98 (7.38)	104.15 (10.56)	100.92 (9.83)	97.46 (8.21)	98.82 (10.32)	98.00 (8.78)	99.19 (6.80)	103.42 (8.67)	101.22 (7.89)	89.29 (8.16)	98.73 (11.68)	94.32 (10.98)
BMI*	25.04 (4.42)	27.75 (5.31)	26.41 (5.05)	25.18 (4.53)	28.34 (6.01)	26.92 (5.56)	23.70 (4.57)	27.14 (5.09)	26.88 (4.61)	25.42 (3.66)	28.98 (4.60)	27.13 (4.44)	21.85 (4.52)	24.77 (3.85)	23.41 (4.30)
WHR*	0.99 (0.05)	0.92 (0.04)	0.95 (0.05)	1.00 (0.04)	0.93 (0.04)	0.96 (0.05)	1.02 (0.06)	0.90 (0.04)	0.97 (0.08)	0.96 (0.03)	0.92 (0.05)	0.94 (0.04)	0.99 (0.07)	0.92 (0.03)	0.95 (0.06)
WHtR*	0.57 (0.05)	0.62 (0.08)	0.59 (0.07)	0.57 (0.06)	0.64 (0.08)	0.60 (0.08)	0.60 (0.04)	0.60 (0.09)	0.60 (0.06)	0.56 (0.03)	0.62 (0.08)	0.59 (0.07)	0.53 (0.07)	0.59 (0.07)	0.56 (0.07)
HOMA-IR*	2.33 (2.96)	3.08 (2.22)	2.71 (2.63)	3.34 (4.13)	3.83 (2.66)	3.61 (3.36)	2.59 (2.81)	2.64 (1.59)	2.61 (2.33)	1.50 (0.75)	2.92 (1.59)	2.18 (1.40)	0.96 (0.54)	1.58 (1.31)	1.29 (1.04)
QUICKI*	0.37 (0.06)	0.34 (0.04)	0.35 (0.05)	0.34 (0.04)	0.32 (0.03)	0.33 (0.03)	0.38 (0.08)	0.34 (0.02)	0.36 (0.07)	0.37 (0.05)	0.34 (0.05)	0.36 (0.05)	0.40 (0.05)	0.38 (0.05)	0.39 (0.05)
HOMA-B*	64.19 (58.76)	84.35 (63.84)	74.37 (61.89)	44.69 (46.38)	48.59 (42.54)	46.84 (43.77)	46.76 (39.43)	83.71 (33.23)	61.54 (40.42)	107.53 (76.04)	147.89 (70.43)	126.90 (74.75)	56.25 (31.69)	87.84 (48.99)	73.10 (43.55)

Table S1: Descriptive population stats and measured clinical parameters summary.

The number of participants (n), males (M), and females (F) are shown in the four classified groups based on HbA1c, along with the parameters observed in the overall studied population. Values are indicated as mean (SD)

***Abbrevations:**

S. Crt.: Serum Creatinine
BMI: Body Mass Index
WHR: Waist to Hip Ratio
WHtR: Waist to Height Ratio
HOMA-IR: Homeostatic Model Assessment of Insulin Resistance
QUICKI: Quantitative Insulin Sensitivity Check Index
HOMA-B: Homeostatic Model Assessment for Beta-cell function

The DNA was amplified using PacBio full length barcoded primers with the following reaction set up and conditions.

Component	Volume
metagenomic DNA	5 µl
Forward Primer (2.5 µM)	3 µl
Reverse Primer (2.5 µM)	3 µl
Nuclease Free Water	1.5 µl
2X KAPA HiFi HotStart ReadyMix	12.5 µl
**Total**	**25 µl**

Table S2. PCR Amplification of V1 – V9 region of 16s gene

S. No.	Temperature	Time	Cycles
1	95°C	3 minutes	1
2	95°C	30 Seconds	32X
3	59°C	30 Seconds	32X
4	72°C	30 Seconds	32X
5	72°C	5 minutes	1
**6**	**4°C**	**Infinite**	**Hold**

Table S3. PCR Amplification Conditions for V1–V9 Region of 16S Gene

Index	Units
Library Preparation Kit	Express Template preparation Kit 2.0
Initial input quantity (PCR Product)	1.7 µg DNA
Shearing	Not Applicable
Final SMRT bell Library Size	1903 bp
Binding Kit	Sequel II Binding Kit 2.1
**Final Library Loading**	**90 pM**

Table S4. Library preparation from the amplified PCR products.
